# Pyrroloquinoline Quinone (PQQ) Inhibits Lipopolysaccharide Induced Inflammation in Part via Downregulated NF-κB and p38/JNK Activation in Microglial and Attenuates Microglia Activation in Lipopolysaccharide Treatment Mice

**DOI:** 10.1371/journal.pone.0109502

**Published:** 2014-10-14

**Authors:** Chongfei Yang, Lifeng Yu, Lingbo Kong, Rui Ma, Juliang Zhang, Qingsheng Zhu, Jinyu Zhu, Dingjun Hao

**Affiliations:** 1 Institute of Orthopaedic Surgery, Xijing Hospital, Fourth Military Medical University, Xi'an, China; 2 Hong-Hui Hospital, Xi'an Jiaotong University College of Medicine, Xi'an, China; 3 Department of Anesthesiology, Xijing Hospital, Fourth Military Medical University, Xi'an, China; National Institute of Allergy and Infectious Diseases - Rocky Mountain Laboratories, United States of America

## Abstract

Therapeutic strategies designed to inhibit the activation of microglia may lead to significant advancement in the treatment of most neurodegenerative diseases. Pyrroloquinoline quinone (PQQ) is a naturally occurring redox cofactor that acts as an essential nutrient, antioxidant, and has been reported to exert potent immunosuppressive effects. In the present study, the anti-inflammatory effects of PQQ was investigated in LPS treated primary microglia cells. Our observations showed that pretreatment with PQQ significantly inhibited the production of NO and PGE2 and suppressed the expression of pro-inflammatory mediators such as iNOS, COX-2, TNF-a, IL-1b, IL-6, MCP-1 and MIP-1a in LPS treated primary microglia cells. The nuclear translocation of NF-κB and the phosphorylation level of p65, p38 and JNK MAP kinase pathways were also inhibited by PQQ in LPS stimulated primary microglia cells. Further a systemic LPS treatment acute inflammation murine brain model was used to study the suppressive effects of PQQ against neuroinflammation *in vivo*. Mice treated with PQQ demonstrated marked attenuation of neuroinflammation based on Western blotting and immunohistochemistry analysis of Iba1-against antibody in the brain tissue. Indicated that PQQ protected primary cortical neurons against microglia-mediated neurotoxicity. These results collectively suggested that PQQ might be a promising therapeutic agent for alleviating the progress of neurodegenerative diseases associated with microglia activation.

## Introduction

Neuroinflammation characterized by the activation of glia has been closely associated with the pathogenesis of a number of neurodegenerative diseases (NDDs), including Alzheimer's disease (AD), Parkinson's disease (PD), amyotrophic lateral sclerosis (ALS) [1]. Microglia, the resident immune cells of the central nervous system, become activated and induce significant and highly detrimental neurotoxic effects by excessively producing a large array of cytotoxic and pro-inflammatory mediators, including inflammatory enzymes such as: inducible Nitric Oxide Synthase (iNOS) and Cyclooxygenase-2 (COX-2); pro-inflammatory cytokines such as: Interleukin-1β (IL-1β) and Tumor Necrosis Factor-α (TNF-α); chemokines such as: Monocyte Chemoattractant Protein (MCP-1), and transcription factors such as: Nuclear Factor-κB (NF-κB) [2]–[4]. Therefore, anti-inflammatory treatment *via* inhibition of microglial activation is regarded as a promising strategy for preventing NDDs in the clinic.

**Figure 1:**
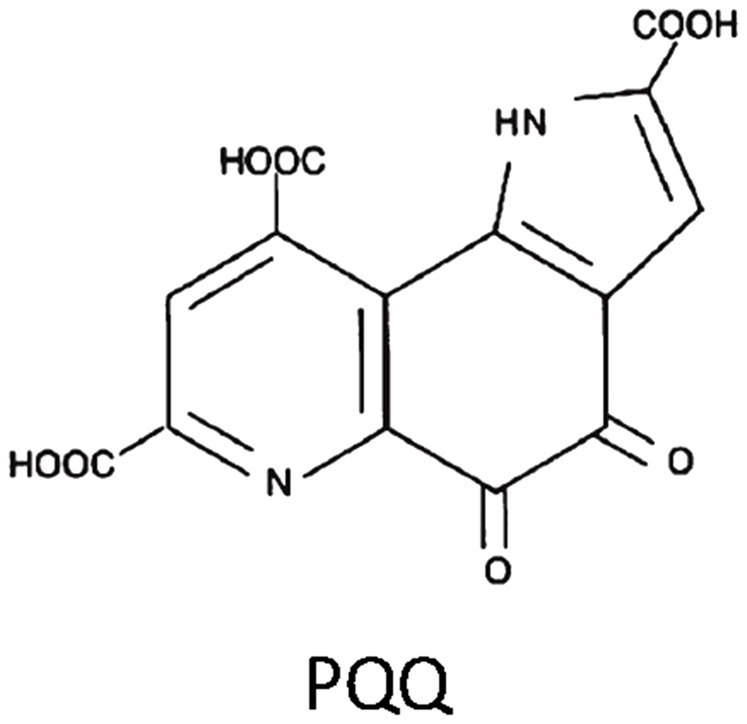
Structure of pyrroloquinoline quinone (PQQ).

Pyrroloquinoline quinone (PQQ) is an anionic, water soluble compound that is a redox cycling planar orthoquinone (Fig. 1) [5], which also has free radical scavenging properties [6], [7]. Only one prior report by Jensen et al. [8] showed that PQQ given intraperitoneally at 30 min prior to hypoxia reduces infarct size without causing measurable neurobehavioral side effects in an *in vivo* cerebral hypoxia/ischemia model in 7-day-old rat pups. Recently PQQ has been reported to depress N-methyl-Dasparate (NMDA)-induced electrical responses and is neuroprotective *in vitro* against NMDA-mediated neurotoxic injury [9]. Further, combined with other previous studies we have shown that PQQ regulate several intracellular signaling pathways, including Ras-related ERK1/2 activation, CREB dependent mitochondriogenesis, and JAK/STAT activation [10]–[12]. However, little information is available about the effects of PQQ on neuroinflammation by using the *in vitro* and *in vivo* study. Therefore, in this study, we aimed to investigate the anti-inflammatory effects of PQQ involved in LPS stimulated mice primary microglia cells activation, and its therapeutic effects on the early stage of neuroinflammation induced by systemic LPS treatment in mice.

## Materials and Methods

Pyrroloquinoline quinine (PQQ) disodium salt was purchased from Wako (Wako Pure Chemical Industries, Ltd. Osaka, Japan). Dulbecco's Eagle's Medium (DMEM), fetal bovine serum (FBS), penicillin and streptomycin, and trypsin/EDTA were purchased from Gibco. LPS from *E.coli* serotype O55:B5 was from Sigma–Aldrich (St. Louis, USA). Antibodies against p38, JNK, phospho-p38, phospho-JNK and NF-κB were purchased from Cell Signaling Biotechnology (Hertfordshire, England). Antibodies against iNOS and COX-2 were from BD Biosciences (Laguna Hills, CA, U.S.A.). Antibody against β-actin and the secondary antibodies were obtained from Santa Cruz Biotechnology (Santa Cruz, CA, U.S.A.). FITC labeled goat anti-rabbit IgG and goat anti-mouse IgG were purchased from Invitrogen (Frederick, MD, USA). Enhanced Chemilumincescence (ECL) kit was from Millipore (Amersham Pharmacia Biotech, Piscataway, NJ). PCR primers were synthesized at Invitrogen (Frederick, MD, USA). The total nitrate assay kit was obtained from Beyotime (Nanjing, China)

### Cell culture

Mice primary microglia cells were prepared from 1 day old C57BL/6J mice as previously described [13]. Briefly, the cerebral cortex was gently dissociated and digested in 0.25% trypsin for 10 min at 37°C. The cells were passed through a 70 µm pore filter and the primary mixed glial cells were resuspended in DMEM supplemented with 10% FBS, 100 U/ml penicillin and 100 mg/ml streptomycin. Cells were seeded in 75 cm^2^ flasks for 10–12 days. Primary microglia cells were separated from the mixed glial cells by shaking the flasks for 3 h at 260 r.p.m. in a rotary shaker at 37°C. Detached cells were cultured in the complete medium and seeded into 24-well plates at a density of 5×10^5^ cells/well for 2–3 days. The purity of the primary microglia cells were more than 95% as determined by Iba-1 staining.

### Cytotoxicity assay for PQQ

Primary microglia seeded in 96-well plates at a density of 5×10^3^ cells/well were treated with PQQ in the presence and absence of LPS for 24 h. Subsequently, MTT solution was added and incubated at 37°C in 5% CO_2_ for 4 h. The dark blue formazan crystals were dissolved in DMSO and the absorbance at 540 nm was determined with a microplate reader. Results were expressed as the percentages of live cells over control cells.

### Measurement of nitrite and PGE2

Primary microglia were seeded at 1.0×10^5^ cells/well in 24-well culture plates, treated with or without PQQ for 1 h followed by LPS treatment (1 µg/ml) for 24 h. Cultured supernatants were collected. Accumulated nitrite was assessed using the Griess reaction. The absorbance was obtained at 570 nm and the results were expressed as mean change fold change of the control. Potassium nitrite was performed as a standard curve. The concentration of PGE2 was measured by a competitive enzyme immunoassay kit (R&D systems, Shanghai, China) following the manufacturer's instructions.

### RNA isolation and real-time quantitative PCR

Total RNA was extracted using Trizol reagent according to the manufacturer's instructions and subsequently reversed transcribed to cDNA using a PrimeScript RT reagent kit. Quantitative PCR was performed using SYBR Premix Ex Taq on an ABI 7500 PCR instrument (Applied Biosystems, USA) as previously described [14]. Relative gene expression was analyzed by the 2^−(△△Ct)^ method with normalization to the expression of the internal control, glyceraldehyde-3-phosphate dehydrogenase (GAPDH). The primers used are as previously described [14].

### Western blot analysis

Equal amounts of protein were separated by a 10% SDS–polyacrylamide gel and transferred to polyvinylidene difluoride (PVDF) membrane. The membrane was blocked with 5% skim milk in TBST for 1 h at room temperature and incubated with primary antibodies against *COX-2* (1∶500), *iNOS* (1∶100), *p38* (1∶1000), *p-p38* (1∶1000), *JNK* (1∶1000), *p-JNK* (1∶1000) over night, then followed by appropriate horseradish peroxidase (HRP)-conjugated secondary antibody (1∶5000), and visualized with the ECL detection kit. The optical density of targeted bands was quantified with densitometry. *β-actin* (1∶5000) was used as a loading control.

### Immunofluorescence analysis

Primary microglia were seeded on cover slips in 24-well culture plates and exposed to LPS (1 µg/ml) for 2 h with or without 1 h PQQ pretreatment. Briefly, cells were washed twice with PBS and fixed in cold 4% parafomaldehyde for 25 min at room temperature. After washing twice in PBS PH 7.4 containing 0.25% Triton X-100, cells were blocked in 3% BSA in PBS for 1 h at room temperature. Subsequently, the cells were incubated overnight with anti-NF-κB p65 in 0.25% PBST and then incubated with FITC-conjugated goat anti-mouse IgG at room temperature for 2 h. DAPI counterstaining was performed to locate the nuclei. Finally, the cover slips were mounted and images were viewed by Olympus BX51 microscope.

### Animal Experiments

Forty-eight healthy female 8-wk-old C57BL/6J mice with a mean weight of 19.9±1.9 g were used in this study. All animal protocols were approved by the Ethics Committee for the Care and Use of Laboratory Animals at the Fourth Military Medical University. The animals were housed in plastic cages at a constant temperature (22±2°C) and humidity (55±10%) with 12 h–12 h light–dark conditions. The animals were allowed free access to food and water before the experiment. A peripheral injection of LPS was administered to evoke neuroinflammation in mice as previously described [15]. Briefly, experimental Groups Mice were randomly divided into four groups. In the sham control group (Normal) mice were injected intraperitoneally (i.p.) with saline (3 mg/kg) was allowed free access to food and water without any treatment. The control group (LPS) was intraperitoneally (i.p.) injected with a single dose of saline (3 mg/kg) and vehicle (normal saline) 1 h before the 1.0 mg/kg LPS (*E.coli*, serotype 055:B5, Sigma) injection. The PQQ treatment groups: LPS+PQQ3 and LPS+PQQ10 were administered two doses of PQQ (3 or 10 mg/kg) once 1 h prior to LPS injection respectively. Their body weights were monitored daily; in a subset of animals, core body temperature was also monitored (DC temperature controller, World Precision Instrument).

### Immunohistochemistry

Microglia activation in the brain tissue was observed with immunohistochemistry. At 4 h after the LPS injection, the mice were deeply anesthetized and perfused transcardially with 0.05 M phosphate-buffered saline (PBS) containing 4% paraformaldehyde. The brain was removed and was post-fixed in the same perfusing solution overnight at 4°C. Coronal sections of 30 µm thickness were made using a freezing microtome (Leica, 2800N, Germany). The brain sections were stained by a free-floating DAB reaction. The sections were rinsed with 0.05 M PBS and then incubated for 15 min in 1% hydrogen peroxide PBS at room temperature. The sections were incubated overnight at 4°C with primary antibodies against Iba-1 (Wako, Japan), as a microglia marker. The sections were then incubated with biotinylated anti-mouse secondary antibody (Vector Laboratories, USA) for 1 h at room temperature, after which the avidinbiotin complex (Vector Laboratories, USA) method was carried out with peroxidase coupling in a mixture containing 0.05% DAB (Sigma-Aldrich, USA) and 0.03% H_2_O_2_ for 2–5 min.

### Count of Iba-1 Immuno-positive Cells

Immunohistochemistry stained sections were used for analysis of the number of the immunopositive cells. The count of Iba-1 immunopositive microglia was analyzed by using ImageJ software (Ver. 1.44p, NIH, U.S.A.). Four sections and four fields per section were chosen for analysis in each rat. Data were normalized with the same area (10^5^ µm^2^) and the mean values for the four sections in each rat were used for statistical analysis.

### Statistical Analysis

Data were expressed as the mean ± S.E.M. of three to five independent experiments. Significant differences were evaluated by ANOVA test followed by *Bonferroni post hoc* multiple comparison test using the SPSS software 18.0. *p-values*<0.05 were considered as statistically significant.

## Results

### Cytotoxicity of PQQ in Primary Microglia

To test the cytotoxicity of PQQ, various concentrations of PQQ were applied alone or together with LPS (1 µg/ml) to primary microglia for 24 h. Cell viability was determined by MTT assay. Normal untreated primary microglia were considered as control. MTT results demonstrated that either treatment of PQQ alone or with LPS had no effect on the cell viability of primary microglia in the performed concentrations. Based on these findings, concentrations of PQQ used in the present study were ranged from 1 to 15 µM.

### PQQ Attenuated LPS-induced NO Production and iNOS Expression in Primary Microglia

**Figure 2:**
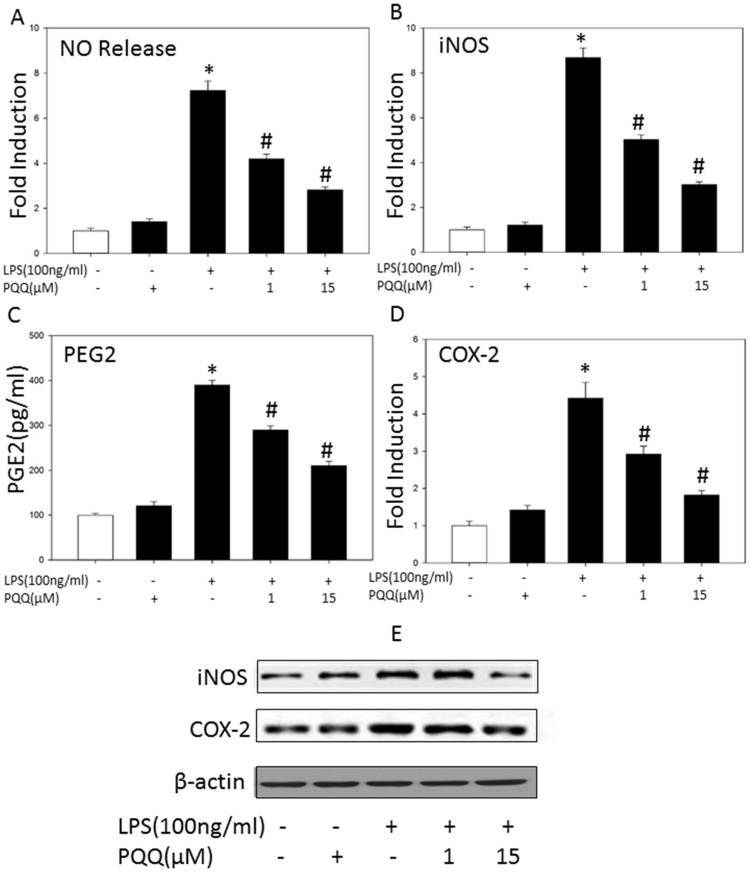
Effects of PQQ on LPS-induced NO, PGE2 production and iNOS, COX-2 expression in microglia cells. Primary microglia were pretreated with or without PQQ for 1 h followed by LPS (100 ng/ml) treatment for indicated durations. Culture supernatants were collected 24 h later, the released NO in primary microglia (A) was determined by the Griess assay, total mRNA was harvested 6 h later, and the mRNA level of iNOS (B) and COX-2 (D) was measured by real-time PCR. (C) Concentrations of PGE2 in the culture supernatants of primary microglia were determined by ELISA. (E) 24 h later, the protein level of iNOS and COX-2 were detected by western blot analysis in primary microglia cells. The results presented as mean ± S.E.M. of at least three independent experiments, *p<0.05 vs. control group, #p<0.05 vs. only LPS group.

To examine the effects of PQQ on NO production in LPS-induced primary microglia, cells were treated with LPS in the presence or absence of PQQ (1, 15 µM) for 24 h and NO levels in the cell culture supernatants were measured. PQQ pretreatment for 1 h effectively decreased LPS-stimulated NO production in a dose-dependent manner in primary microglia cells (Fig. 2A). Subsequently, we further determined the iNOS mRNA and protein levels by real-time PCR and western blot in primary microglia cells respectively. Consistent with the down-regulation of NO production, PQQ pretreatment significantly attenuated LPS-induced iNOS mRNA (Fig. 2B) and protein levels (Fig. 2E) at 15 µM in primary microglia cells.

### PQQ Suppressed LPS-induced PGE2 Production and COX-2 Expression in Primary Microglia

To examine the effect of PQQ on PGE2 production in LPS-induced primary microglia, cells were treated with or without PQQ following the treatment of LPS for 24 h. Concentrations of PGE2 in the culture supernatants were measured by ELISA. As shown in Fig. 2C, LPS-stimulated PGE2 production was markedly decreased by PQQ pretreatment in a dose-dependent manner in primary microglia. To further examine whether the down-regulation of COX-2 contributed to the decrease in PGE2 production, real-time PCR and western blot analysis were performed to analyze the expression of COX-2. Treatment with PQQ 1 h prior to LPS stimulation significantly suppressed both LPS-induced COX-2 mRNA (Fig. 2D) and protein levels (Fig. 2E) at 15 µM. Results demonstrated that inhibition of COX-2 expression by PQQ pretreatment was probably associated with the reduction of PGE2 production in LPS-induced microglia.

### PQQ downregulated LPS-induced mRNA levels of pro-inflammatory Mediators in Primary Microglia

**Figure 3:**
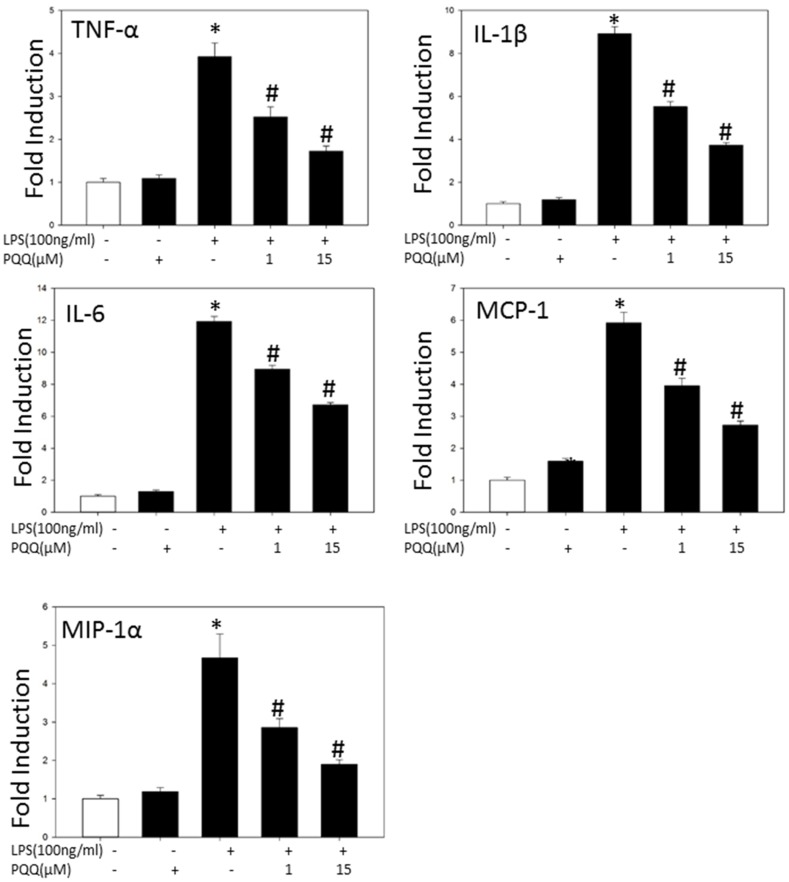
Effects of PQQ on LPS-stimulated expression of pro-inflammatory mediators in microglial cells. Cells were treated with the indicated concentrations of PQQ 1 h prior to 6 h co-treatment of LPS (100 ng/ml). Primary microglia were harvested and total RNA was prepared. The mRNA expression of pro-inflammatory mediators: TNF-a, IL-1β, IL-6, COX-2, MCP-1 and MIP-1α was measured by real-time PCR. GAPDH was used as an internal control. The results shown are mean ± S.E.M. of three independent experiments. *p<0.05 vs. control group, #p<0.05 vs. only LPS group.

To examine the effect of PQQ on LPS-induced mRNA overexpression of pro-inflammatory cytokines and chemokines, primary microglia cells were treated with PQQ (1 µM, 15 µM) for 1 h prior to stimulation with LPS for 6 h. Results of real-time PCR show the mRNA expression of pro-inflammatory factors in primary microglia cells was all dramatically increased in response to LPS stimuli compared to the control. However, the production of these pro-inflammatory factors was significantly reduced in a dose-dependent manner by pretreatment with PQQ. These results indicated that LPS-induced excessive mRNA expression of TNF-α, IL-1β, IL-6, MCP-1 and MIP-1α were effectively down-regulated by PQQ pretreatment (Fig. 3).

### PQQ Inhibited LPS-induced Activation of NF-κB in Primary Microglia

**Figure 4:**
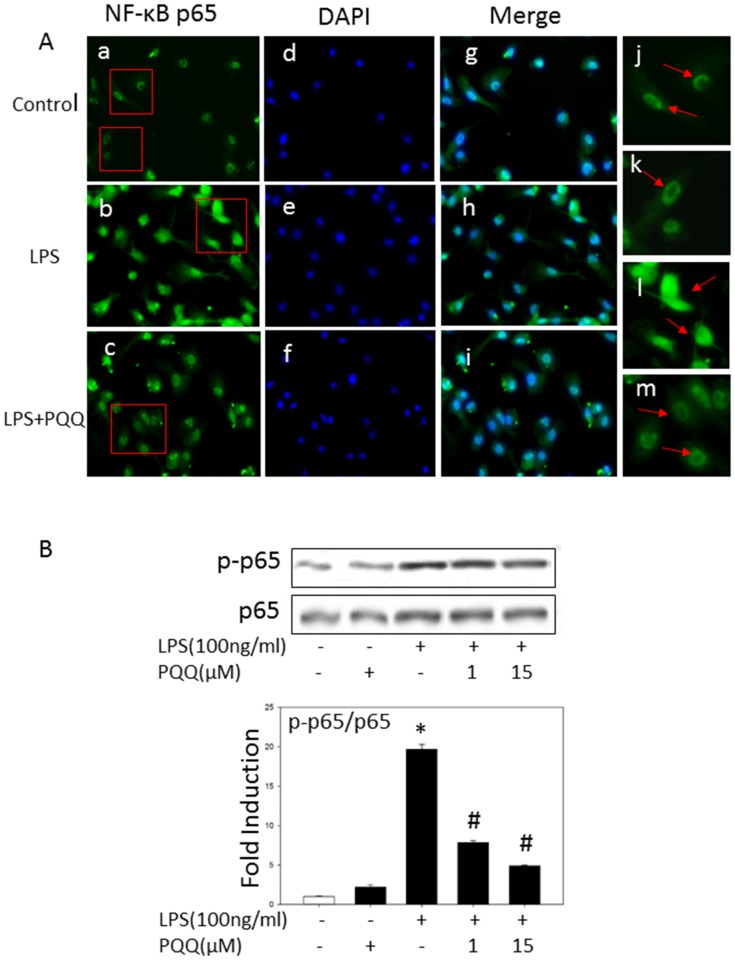
Effects of PQQ on LPS-induced p65 and NF-κB activity in microglial cells. (A) Representative images of NF-κB p65 in microglial cells of each group. Cells were pretreated with or without PQQ for 1 h followed by 100 ng/ml LPS treatment for 2 h. Microglial cells were incubated with NF-κB p65 antibody and immunofluorescence microscopy was used to visualize the localization of NF-κB p65 (Green; a-c), boxed regions in (a-c) are also shown at×200 (j-m). Nuclei were visualized using DAPI counterstaining (Blue; d-f). (B) Cells were treated with 100 ng/m LPS for indicated time. p65 protein level was measured by western blot analysis. Non-phosphorylated p65 was used as loading control, and the expression of p-p65 was normalized to control and quantified by densitometric analysis. The results shown are mean ± S.E.M. of three independent experiments. *p<0.05 vs. control group, #p<0.05 vs. only LPS group.

NF-κB is an important regulator of gene expression of pro-inflammatory mediators such as COX-2, iNOS, TNF-α and IL-6 in LPS-induced microglia. Therefore, we identified the effects of PQQ on NF-κB activity using immunofluoresence microscopy. As shown in Fig. 4A, LPS exposure induced significant nuclear translocation of NF-κB p65 subunit in primary microglia. In contrast, PQQ pretreatment strongly inhibited this response. These results indicated that PQQ may attenuate NF-κB activation through inhibiton of NF-κB p65 nuclear translocation.

### PQQ Attenuated the Phosphorylation Levels of p38 and JNK in LPS-treated Primary Microglia

**Figure 5:**
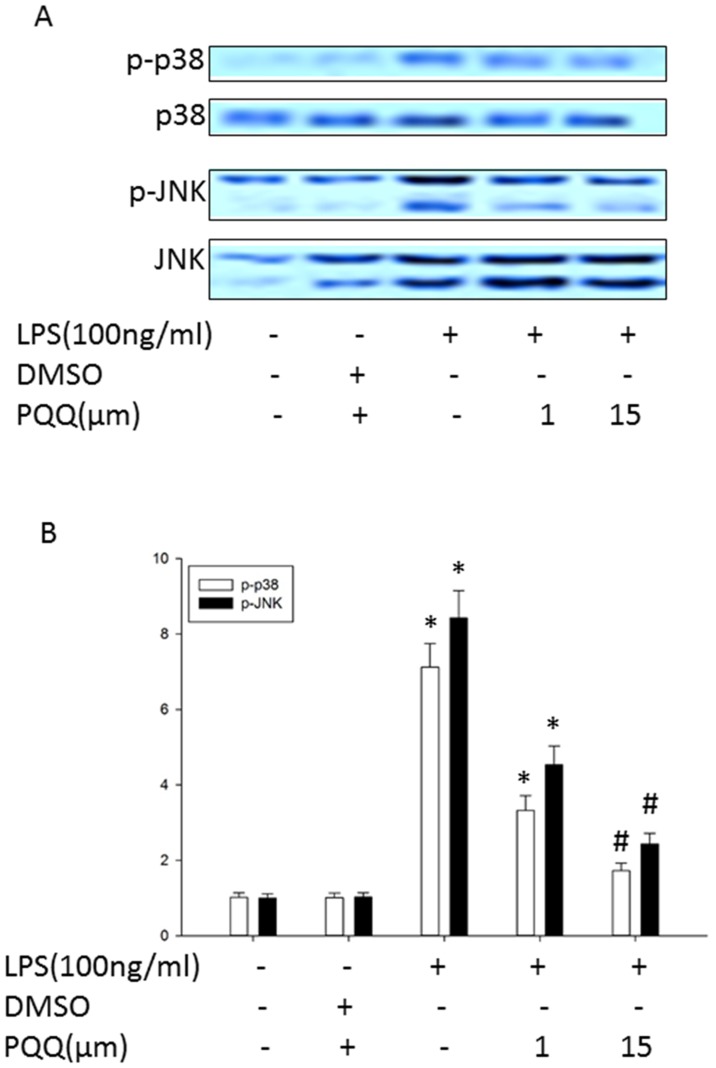
Effects of PQQ on LPS-induced MAP kinases activity in microglial cells. (A) Microglial cells were treated with or without PQQ 1 h prior to 4 h co-treatment with LPS. The protein expression of p-p38 and p-JNK was determined by western blot analysis. Non-phosphorylated form of each targeted protein was used as loading control. (B) Bar graph represents the average levels of p-p38 and p-JNK compared to the control. The results shown are mean ± S.E.M. of three independent experiments. *p<0.05 vs. control group, #p<0.05 vs. only LPS group.

MAP kinases signaling is one of the most important pathway in modulating inflammatory activation in microglia [16]. Therefore, we also studied the role of PQQ in LPS-induced activation of MAP kinases pathways in primary microglia. As shown in Fig.5, LPS stimulation led to rapid activation of p38 and JNK, then cells were treated with or without PQQ for 4 h. Western blot analysis showed that PQQ pretreatment significantly inhibited LPS-stimulated phosphorylation of p38 and JNK while no changes were observed in the expression of non-phosphorylated p38 and JNK. These results indicated that inhibition of pro-inflammatory mediators by PQQ in LPS-induced primary microglia was partly associated with down-regulation of the activity of p38 and JNK.

### PQQ Attenuates Microglia Activation in the Brain Tissue of LPS-Treated Mice

**Figure 6:**
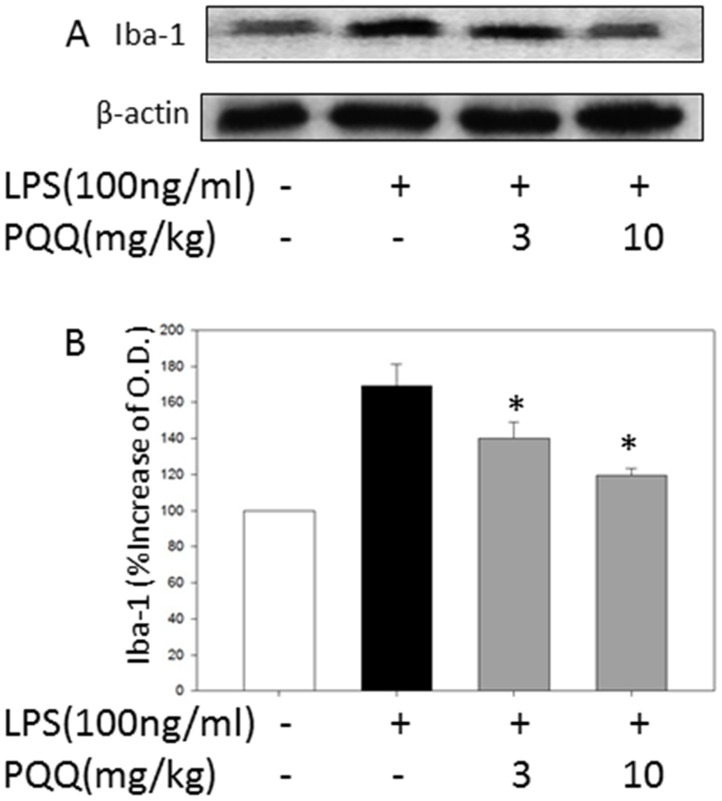
Effect of PQQ on Iba-1 Expression in the Brain. Representative Western blots illustrate differences in the bands of Iba-1 (A). LPS up-regulates Iba-1 expression in brain tissue, while PQQ treatment attenuates up-regulation of Iba-1 expression at doses of 3 and 10 mg/kg administration (B). The results shown are mean ± S.E.M. (*n* = 6 in each group) of three independent experiments. *p<0.05 vs. control group, #p<0.05 vs. only LPS group.

The effects of PQQ on microglia activation following systemic LPS injection were tested by quantification of Iba-1 protein with Western blotting and immunohistochemistry against Iba-1 antibody in the brain tissue at 4 h after LPS injection. LPS increased the % increase of Iba-1 expression in brain tissue (169.20±11.97%). PQQ treatment attenuated, significantly and dose dependently, the % increase of Iba-1 expression in the brain tissue at doses of 3 and 10 mg/kg (139.91±9.29%, *p<0.05*; 119.37±3.93%, *p<0.05*; respectively) as compared to the LPS group (Fig.6).

**Figure 7:**
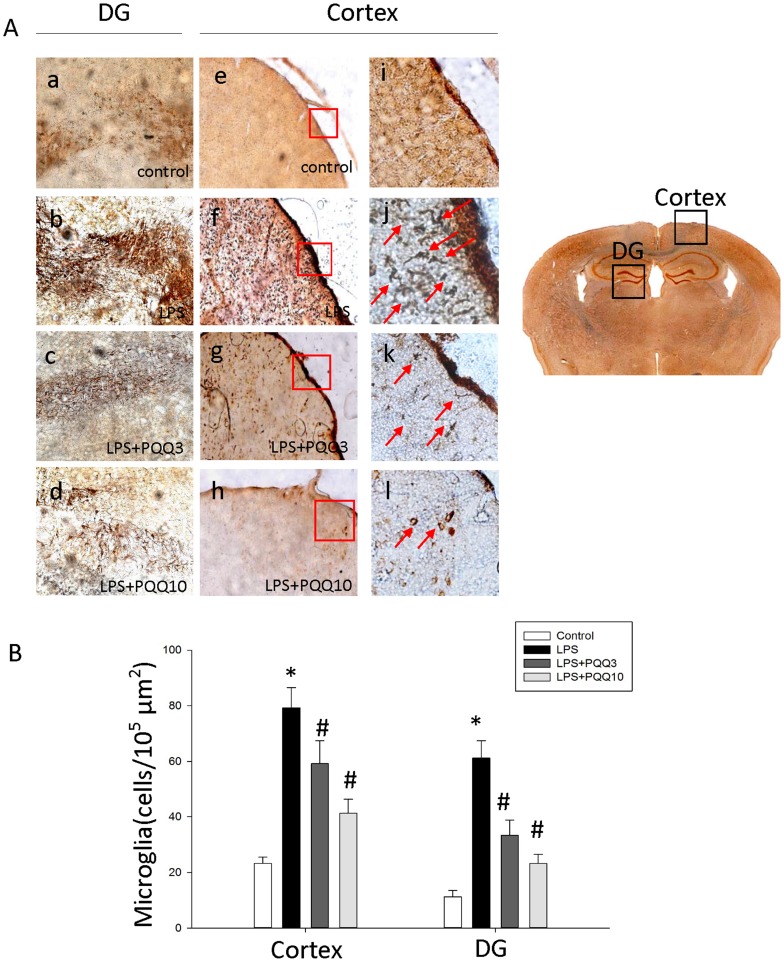
Effect of PQQ on Microglia Activation in the Brain. Representative photographs show Iba-1 immuno-stained microglia of the mouse brain (A). LPS group shows mostly an activated-form of microglia which display an increased size, irregular shape, thickened and shortened processes and intensified Iba-1 immunostaining density, in the dentate gyrus of hippocampus (DG) and cerebral cortex (Cortex) compared to the normal group. Moreover, both PQQ treated groups show a decrease of morphological activation of microglia in all brain regions with respect to LPS group. Representative DG (a–d) and Cortex (e–h) immunostained slices are presented at ×40. High power images of Iba-1-expressed microglia in the boxed regions in (f), (g) and (h) are also shown at×200 (i–l). Arrow indicates the Iba-1-expressed microglia. The number of microglia was counted and normalized in the corresponding same area (B). LPS increases the number of Iba-1-expressed microglia in the brain. PQQ treatment significantly reduces the number of Iba-1-expressed microglia both in the cerebral cortex and DG. The results shown are mean ± S.E.M. (*n* = 6 in each group) of three independent experiments. *p<0.05 vs. control group, #p<0.05 vs. only LPS group.

The LPS injection activated microglia, as displayed by an increase in cell size, irregular shape, thickened and shortened processes and intensified Iba-1 immunostaining density in the cerebral cortex and dentate gyrus (DG) of hippocampus (Fig. 7A). However, compared with the LPS group, PQQ treatment dramatically attenuated the morphological changes to activated-form of microglia. To examine the effect of PQQ on microglia activation, the number of Iba-1 immunostained microglia was counted and normalized with the same area (10^5^ µm^2^). In the cerebral cortex area, the LPS group was 78.7±7.9 cells/10^5^ µm^2^. However, the PQQ treatment groups were 58.9±6.9 cells/10^5^ µm^2^ (p<0.05) and 41.3±3.9 cells/10^5^ µm^2^ (p<0.05) respectively (Fig. 7B). In the dentate gyrus (DG) area, the LPS group was 64.1±6.19 cells/10^5^ µm^2^. However, the PQQ treatment groups were 36.3±3.3 cells/10^5^ µm^2^ (p<0.05) and 27.7±2.9 cells/10^5^ µm^2^ (p<0.05) respectively (Fig. 7B). Those results indicate that PQQ attenuated microglia activation in the brain induced by systemic LPS injection.

## Discussion

The present study demonstrated for the first time that PQQ attenuated the synthesis of NO and PGE2, suppressed the expression of iNOS and COX-2 at the mRNA and proteins levels and inhibited the mRNA levels of TNF-α, IL-1β, IL-6, MCP-1 and MIP-1α in LPS-stimulated primary microglia cells. The nuclear translocation of NF-κB and the activation of p38 MAP kinase and JNK were also suppressed by PQQ *in vitro*. Moreover, PQQ exhibits suppressive effects against neuroinflammation induced by systemic LPS treatment *in vivo*. These data suggested that PQQ would be a potential therapeutic agent for alleviating neuroinflammation desease accompanied by activated microglia.

Microglia-mediated neurotoxicity is a hallmark of the pathogenesis of various neurodegenerative diseases [17]. High levels of pro-inflammatory cytokines and chemokines released by activated microglia are implicated in the process of neuronal injury [18]. Many recent studies have reported that agents exert their neuroprotective effects through inhibition of production of pro-inflammatory mediators [19]–[22]. Moreover, excessive activation of microglia characterized by robust production and secretion of a variety of pro-inflammatory substances has been implicated in the pathogenesis of a number of neurodegenerative diseases, such as AD and PD [23]. Accumulation of these pro-inflammatory molecules leads to neurodegeneration and eventually neurological diseases [24]. Thus, therapeutic approaches targeting activated microglia may be a promising treatment of these diseases. Despite major roles of microglia on inflammatory responses in the brain, there were no reports to demonstrate effects of PQQ on microglia activation *in vitro* or *in vivo*.

COX-2 is upregulated in response to various inflammatory stimuli including overproduction of PGE2. Up-regulation of COX-2 contributes to the development of many chronic inflammatory diseases [25], [26]. Moreover, NO also is an important regulatory mediator involved in cell survival and death exerts a number of pro-inflammatory effects during several physiological and pathological processes. Prolonged activation of microglial cells leads to excessive release of NO by iNOS in the brain [27]. Under conditions in neurodegenerative diseases, increased production of NO mainly results from the up-regulated expression of iNOS in activated astrocytes and microglia cells [28]. Therefore, agents that inhibit the release of NO and attenuate iNOS and COX-2 expression could be beneficial for preventing and delaying the progression of neuroinflammatory disease. In the present study, PQQ treatment to primary microglia stimulated by LPS effectively decreased iNOS and COX-2 levels and the release of their respective end-products, NO and PGE2. This inhibition exhibited by PQQ may be attributed to suppression of iNOS and COX-2 mRNA transcription and a subsequent reduction in protein expression.

The transcription factor NF-κB is a pleiotropic regulator of diverse genes involved in regulating the expression of various pro-inflammatory mediators and has been found to be a promising target for inflammatory diseases [29]. NF-κB activates several cellular signal transduction pathways that are involved in the production of iNOS, COX-2, and various cytokines [30]–[32]. As the expression of these pro-inflammatory mediators is modulated by NF-κB, blocking NF-κB transcriptional activity may be an important target for treating inflammatory diseases. Therefore in this study, we investigated the inhibition effects of PQQ on NF-κB activity. We found that PQQ pretreatment markedly suppressed LPS-induced nuclear translocation of NF-κB p65, suggesting that inhibition of pro-inflammatory factors by PQQ was at least partially mediated through blockade of NF-κB activation.

MAP kinases are also crucial in regulating the pro-inflammatroy substances such as TNF-α, IL-6, iNOS and COX-2 expression in LPS-stimulated microglia cells [33], [34]. One of the important MAPK families, p38, is positively related to LPS signaling in microglial cells, which respond to pro-inflammatory cytokines [16]. Moreover, p38 MAPK is a key mediator of cellular stressors such as inflammation and apoptosis. Both *in vitro* and *in vivo* studies have shown that p38 MAPK regulates the production of the pro-inflammatory cytokines, NO, and PGE2 by increasing cytokine release or messenger RNA transcription [35], [36]. Thus, we further determined whether the anti-inflammatory effects of PQQ in LPS-stimulated primary microglia was due to suppression of MAP kinases. Results from our study shown that exposure of primary microglia to LPS resulted in strongly increased expression of phosphorylated p38 and JNK, indicating that p38 and JNK are additional pathways by which PQQ inhibited LPS-induced inflammatory activation of primary microglia.

At the *in vivo* experiment, we used the systemic LPS treatment acute inflammation murine brain model to study the suppressive effects of PQQ against neuroinflammation. Microglia are activated in response to brain injuries and immunological stimuli to undergo dramatic alterations in morphology, changing from resting, ramified microglia into activated, amoeboid microglia, which is believed to favor phagocytosis and mobility [37]. In our present *in vivo* study, according immunohistochemical analyses, we found PQQ lessened the microglia morphological changes to an activated-form which can manifest as increased cell size, irregular shape, thickened and shortened processes in all brain regions. Moreover, PQQ significantly reduced Iba-1 protein expression in the brain compared to that of LPS group. Iba-1, which is highly and specifically expressed in microglia and macrophage [36], and is involved in RacGTPase-dependent membrane ruffling and phagocytosis [38]. By immunohistochemical analyses, anti-Iba-1 antibody was found to specifically recognize ramified microglia in normal rat brain, and Iba-1 protein was strongly upregulated in activated microglia. Therefore, Iba-1 may play significant roles in the regulation of some immunological and pathophysiological functions of microglia, and serve as a novel marker of detecting the activation of microglia. However, this study is the first time to show that PQQ plays a modulatory role in microglia activation using an *in vivo* animal study.

In conclusion, PQQ showed significant anti-neuroinflammatory actions in microglial cells regulating *via* NF-κB and p38 MAPK signaling pathways *in vitro*. Further, the *in vivo* efficacy of PQQ was confirmed with an animal model of systemic LPS treatment acute inflammation murine brain model. Given that microglial activation and the consequent release of various inflammatory components contributes to neurodegeneration, PQQ may potentially protect against microglia-mediated neurodegenerative diseases. Further in-depth and long term research is required to confirm these findings.
